# Boron-containing delocalised lipophilic cations for the selective targeting of cancer cells†
†The authors declare no competing interests.


**DOI:** 10.1039/c6md00383d

**Published:** 2016-10-28

**Authors:** Calabrese Gianpiero, Daou Anis, Rova Aikaterini, Tseligka Eirini, Vizirianakis S. Ioannis, Fatouros G. Dimitrios, Tsibouklis John

**Affiliations:** a School of Life Science, Pharmacy and Chemistry , Kingston University London , Penrhyn Road , Kingston-upon-Thames , Surrey KT1 2EE , UK . Email: G.Calabrese@kingston.ac.uk; b Department of Pharmacology , School of Pharmacy , Aristotle University of Thessaloniki , GR-54124 Thessaloniki , Greece; c Department of Pharmaceutical Technology , School of Pharmacy , Aristotle University of Thessaloniki , GR-54124 Thessaloniki , Greece; d School of Pharmacy and Biomedical Sciences , University of Portsmouth , Portsmouth , PO1 2DT , UK

## Abstract

To limit the incidence of relapse, cancer treatments must not promote the emergence of drug resistance in tumour and cancer stem cells. Under the proviso that a therapeutic amount of boron is selectively delivered to cancer cells, Boron Neutron Capture Therapy (BNCT) may represent one approach that meets this requirement. To this end, we report the synthesis and pharmacology of several chemical entities, based on boron-rich carborane moieties that are functionalised with Delocalized Lipophilic Cations (DLCs), which selectively target the mitochondria of tumour cells. The treatment of tumour and cancer stem cells (CSCs) with such DLC-functionalized carboranes (DLC-carboranes) induces cell growth arrest that is both highly cancer-cell-selective and permanent. Experiments involving cultures of normal and cancer cells show that only normal cells exhibit recapitulation of their proliferation potential upon removal of the DLC-carborane treatment. At the molecular level, the pharmacological effect of DLC-carboranes is exerted through activation of the p53/p21 axis.

## Abstract 2

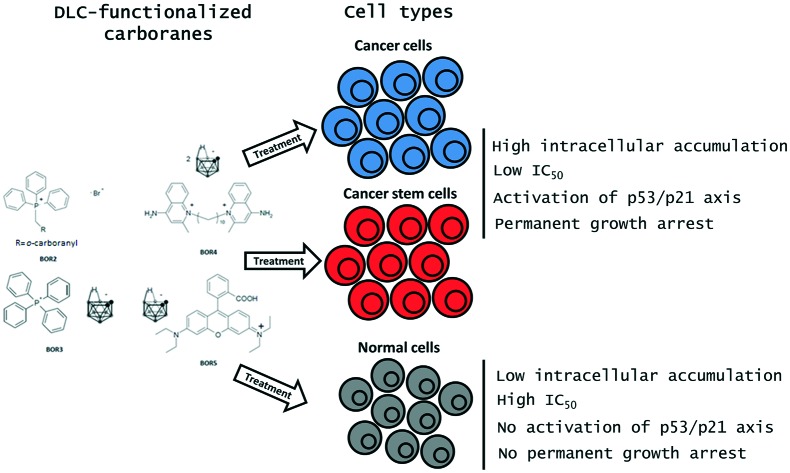
To limit the incidence of relapse, cancer treatments must not promote the emergence of drug resistance in tumour and cancer stem cells.

## Introduction

It is predicted that by the end of 2016 there will be 78 000 new cases of primary brain tumours; this figure includes nearly 25 000 primary malignant and 53 000 non-malignant brain tumours.^1^ Consequent to these levels of incidence, around 17 000 people will lose their battle against primary malignant and central nervous system (CNS) brain tumours.^2^ Glioblastomas, which are categorised by the World Health Organization according to increasingly unfavourable prognosis as grades I to IV, account for 55% of all gliomas and 15% of all primary brain tumours.^3^

Cancer cell initiation and progression represent complex multifactorial processes that impede the efficacy and safe clinical outcome of pharmacological interventions. Tumour cells' heterogeneity and microenvironment present major challenges at the clinical setting. Cancer treatment protocols are determined by location, type and stage. Most commonly, preliminary surgery is employed to remove as much tumour tissue as possible, followed by radiotherapy and/or chemotherapy.^4^ Since glioblastomas possess a high metastasising capability, relapse rates are very high.^5^ Recent experimental evidence has identified cancer stem cells (CSCs) as a subpopulation that exhibits distinct cellular and genomic characteristics that render these cells capable of escaping radiotherapy- and chemotherapy-induced cell death by staying arrested over time at the G_1_/G_0_ cell growth phase, which in turn allows them to re-enter the cell cycle under appropriate microenvironment conditions. This implies that research efforts have to be focused on the design of anti-cancer molecules, the therapeutic range of which includes CSCs.^6^–^8^ Owing to the capability of recently developed BNCT agents to target cancer cells, including CSCs, both efficiently and selectively, the clinical use of these agents may be characterized by low incidence drug resistance profiles.^9^,^10^

Apart from the phenotypic distinctions, several differences between mitochondria in normal and in tumour cells have been observed, and some have been rationalized at the genetic, molecular and biochemical levels.^4^,^11^,^12^ ATP synthesis *via* oxidative phosphorylation displays an electrochemical gradient that incorporates contributions from the pH gradient and the potential difference across the inner membrane of mitochondria.^13^,^14^ The *in vitro* mitochondrial trans-membrane potential of a healthy cell is in the range 180–200 mV; whereas, the corresponding *in vivo* potential is of the order of 130–150 mV.^15^ The mitochondrial trans-membrane potential of carcinoma cells is *ca.* 60 mV higher than that of epithelial cells.^16^ This difference makes mitochondria promising targets for selective drug delivery. In addition, the mitochondria of CSCs or tumour-initiating cells (TICs) exhibit features – *e.g.* increased glycolytic metabolism, different redox state regulation (mitochondrial membrane potential, expression of apoptosis-related proteins) – that distinguish them from the ordinary, more differentiated tumour cells. These features may be exploited to guide the design of therapeutics that target cancer cells with a high degree of specificity.^17^,^18^

## Boron neutron capture therapy (BNCT)

**Fig. 1:**
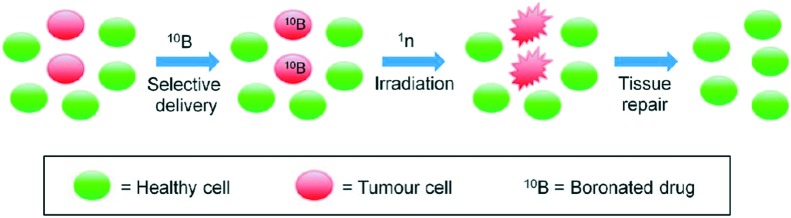
BNCT steps: the selective delivery of ^10^B-containing drugs to tumour cells is followed by irradiation with thermal neutrons (^1^n) to initiate the destruction of cancer cells and to allow tissue repair.

BNCT is a two-step chemo-radiotherapeutic technique (Fig. 1) that involves the selective delivery of ^10^B-rich agents to tumours and their subsequent irradiation with low-energy neutrons.

The interaction of ^10^B with low-energy neutrons (*i.e.* the capture reaction) results in nuclear fission that effects the selective destruction of the host tumour cells. During this reaction, energetic alpha particles possessing high linear energy transfer (LET), low oxygen enhancement ratio and high relative biological effectiveness are produced. LET particles are lethal but – because of their size, energy and short path lengths (4.5–10 μm) – the effect is confined to the host cell.^19^ The predominant products of the ^10^B neutron capture reaction are recoiling ^7^Li nuclei and α particles. It takes only a few α particles, which are as lethal to hypoxic and oxygenated cells as they are to non-proliferating cells, to kill a malignant cell.^20^ If ^10^B is accumulated selectively in cancer cells and, assuming that in surrounding healthy tissue its concentration does not exceed a certain critical value, the cytocidal effects of the capture reaction are limited to malignant cells. Inevitable capture reactions involving ^1^H and ^14^N from normal tissue, which respectively produce γ rays and protons, are of little relevance since the corresponding thermal-neutron-capture cross-sections for these nuclei are too small to induce marked complications during BNCT.^21^

BNCT has been investigated for use in the treatment of glioblastoma multiforme (GBM),^22^ a malignancy with a current mean survival time of <12 months.^23^ BNCT offers promise in GBM, but significant research effort is required before the many performance requirements for successful treatment are met,^24^ including the synthesis of ^10^B-enriched compounds with very low inherent toxicity and integration of the selective targeting strategy into the molecular design, such that therapeutically useful concentrations (>10^9^) of ^10^B atoms/tumour cells are achieved while a tumour/blood concentration ratio >5 : 1 and a tumour/normal tissue concentration ratio >3 : 1 are maintained throughout the neutron capture stage of the treatment.

The multitude of performance demands that ^10^B-containing drugs need to satisfy before they can be used in the clinic is reflected by the very small number of ^10^B compounds that have reached this stage: *para*-boronophenylalanine (BPA) and sodium mercaptoundecahydrododecaborate (BSH), which respectively incorporate 1 and 12 boron atoms in their molecular structures. Following an early attempt by Pitochelli and Hawthorne^25^ and fuelled by the data of clinical trials conducted by Hatanaka^26^ in Japan, polyhedral boranes have become the materials of choice for the delivery of ^10^B-rich moieties to cancer cells. Among the common polyhedral boron agents, the neutral lipophilic icosahedral dicarba-*closo*-dodecarboranes (C_2_B_10_H_12_; commonly referred to as carboranes), which also exist as *ortho*-, *meta*- and *para*-isomers, are of particular interest not only because of their high ^10^B content, good catabolic stability and low toxicity, but also because of their amenability to chemical functionalisation.^21^ The base-induced removal of one boron atom from the icosahedral cage transforms the lipophilic *closo*-carborane into the corresponding *nido*-carborane, which is more hydrophilic and hence more compatible with water-based systems.

## Mitochondrial targeting

**Fig. 2:**
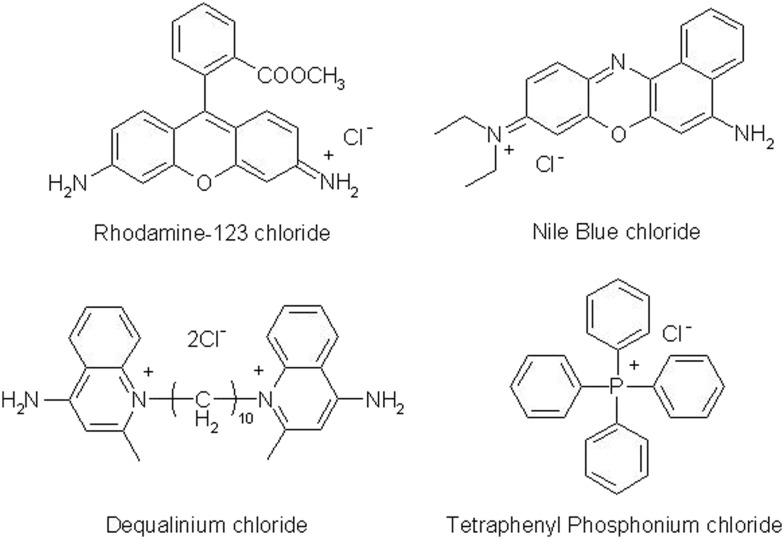
Typical delocalized lipophilic cations (DLCs): rhodamine-123 chloride, nile blue chloride, dequalinium chloride and tetraphenylphosphonium chloride.

Integral to the performance requirements for BNCT is the molecular design of boron compounds that are capable of targeting intra-cellular organelles. Neoplastic cells are characterized by high metabolic activity, which renders the design of therapeutic agents that target the cellular organelles possible, of both dividing and non-dividing cancer cells.^27^ Since the negative-inside transmembrane potential of mitochondria (130–150 mV) is far greater than that of any other organelle, functionalisation with DLC moieties represents a means of imparting mitochondrial targeting specificity to carboranes.^28^ Some examples^29^,^30^,^31^,^32^ of DLCs that have been shown to accumulate preferentially in malignant cells are presented in Fig. 2.

As the name implies, all DLCs are amphiphilic cationic compounds in which the positive charge is delocalized over an extensive π electron system. Their lipophilic nature and the delocalization of the positive charge over a large area act cooperatively to reduce the free energy change when DLCs diffuse through lipid membranes, such as those of mitochondria. Driven by the mitochondrial membrane potential, the accumulation of DLCs may be described by the Nernst equation:Δ*Ψ*(mV) = 61.5 log_10_{[cation]_in_/[cation]_out_}

Since the mitochondrial membrane potential of carcinoma cells is *ca.* 60 mV greater than that of normal epithelial cells,^16^ one consequence of the Nernstian relationship is that there is a ten-fold increase in the propensity of DLCs to accumulate within the mitochondria of such cells.^33^ This process is further assisted by the plasma membrane potential (typically 30–60 mV; negative inside), which promotes the increased accumulation of cations into carcinoma cells prior to their localisation into mitochondria. The synergistic effect results in 90–95% of the available intracellular cations becoming localised at mitochondrial sites.^34^

DLCs, often termed mitochondriotropics,^35^,^36^ are used extensively in the visualisation of mitochondria and also in the estimation of mitochondrial activity.^37^,^38^ The use of these materials in diagnosis is constrained only by their concentration-dependent toxicity to mitochondria. The toxic effects of rhodamine-123 and dequalinium chloride, two of the most commonly used DLCs, have been linked to their capacity to inhibit the enzymes F0F1 ATPase^39^ and NADH-ubiquinone reductase.^40^ Oral administration to mice bearing transplanted tumours has shown that substances containing a benzo[*a*]phenoxazine nucleus, such as Nile blue chloride, exhibit a tumour-staining and growth-retarding action.^41^ Studies have shown that the subcellular localisation of Nile blue chloride is highly selective to lysosomal targets and also that cellular uptake in tumours involves an ion-trapping mechanism.^42^ Also, DLCs have been shown to cause mitochondrial depolarisation, which leads to the opening of mPTPC and the consequent loss of pyridine nucleotides from the mitochondrial matrix. This effects a further increase in mitochondrial membrane potential,^43^ which promotes the influx of DLCs into cancer cells. Such cells exhibit mitochondrial abnormalities that include progressive swelling, disruption of the mitochondrial cristae, concomitant mitochondrial outer membrane rupture and multiple mitochondrial lesions, which ultimately result in cell death.^44^

## Boronated DLCs

Although DLCs are promising carriers for the selective tagging of ^10^B to mitochondria of cancer cells, there are very few literature examples of synthetic compounds that combine boronated entities with DLC moieties such that their corresponding therapeutic effect and targeting specificity are combined in a single molecular structure.

**Fig. 3:**
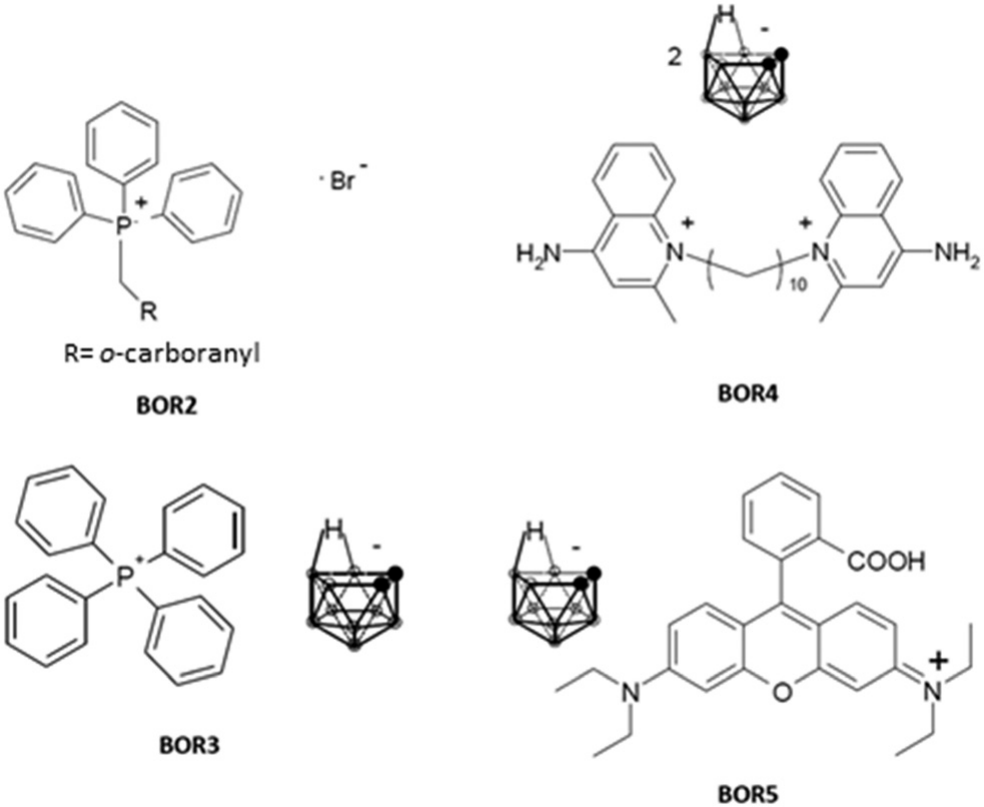
DLC-functionalised carboranes: triphenyl methylenecarboranyl phosphonium bromide (BOR2) and *nido*-carborane salts of tetraphenyl phosphonium (BOR3), dequalinium (BOR4) and rhodamine-B (BOR5).

Adams *et al.*^45^ synthesised a carboranyl derivative of dequalinium (DEQ-B), which has been shown by *in vitro* evaluations to exhibit tumour uptake and toxicology that are similar to those characterising its non-boronated analogue. *In vitro*, DEQ-B was seen to be taken up and retained by KB, F98, and C6 tumour cell lines, but not by the normal epithelial cell line CV1. At low concentrations, DEQ-B was shown to be less toxic towards the latter cell line. The uptake, retention and toxicity of DEQ-B were found to be comparable with those of other non-boronated DLCs, such as dequalinium chloride, rhodamine 123 and tetraphenylphosphonium chloride.^45^ Another example of a boronated DLC derivative is rhodamine B-phenyl boronic acid (containing a single ^10^B atom per molecule), which has been employed as a fluorescent marker and has been shown to accumulate selectively in colon carcinoma cells at a cancer/normal cell ratio of 5.5.^46^ Calabrese *et al.*^47^ reported the synthesis of a series of compounds in which carboranes are combined with DLCs. A preliminary *in vitro* evaluation study, utilising human prostate carcinoma (PC3) and normal (PNT2) epithelial cell lines, indicated the combined propensity of these agents to target tumour cells and to deliver therapeutically relevant quantities of boron.^48^ Consistent with the increased mitochondrial membrane potential of carcinoma cells, the percentage of ^10^B taken up by PC3 cells was higher than that determined in parallel experiments involving PNT2 cells. The data indicated that, with the exception of one Nile blue derivative, the synthesized compounds exhibited features such as uptake efficiency, tumour selectivity and capability to deliver therapeutically relevant amounts of boron that are pre-requisite to candidate materials for BNCT of cancer. Fig. 3 presents the structures of compounds selected for further evaluation.^10^ Rendina *et al.*^48^ reported the synthesis of *closo*-carborane phosphonium salt derivatives and commented on the absence of a covalent bond between the anionic boron entity and the cation in the compounds reported by Calabrese *et al.*^47^*In vitro* boron uptake studies have shown^48^ that advantageous cancer/healthy cell distribution ratios and overall levels of boron accumulation can be achieved irrespective of the presence or absence of a covalent bond between the carborane and DLC moieties. Hence, it appears that a covalent link between the DLC and the boron moiety may not be necessary for the selective delivery of boron to tumour sites.^49^

**Fig. 4:**
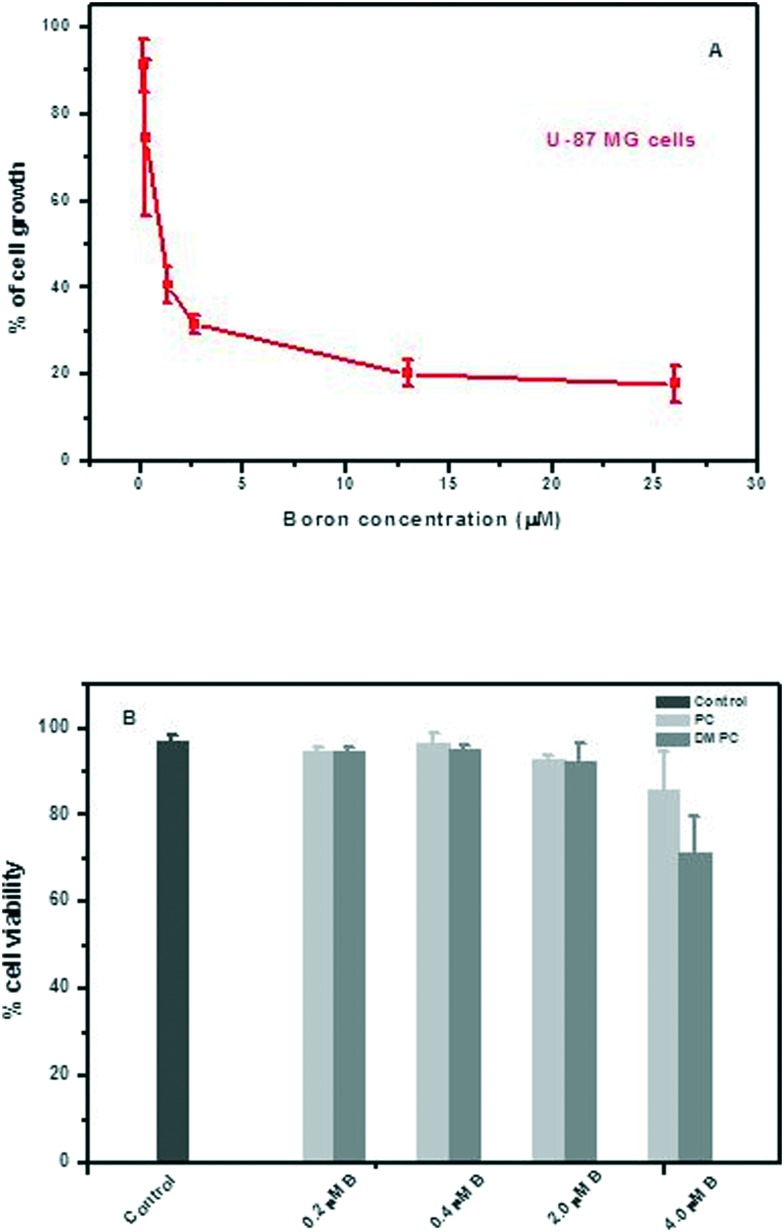
(A) Cell growth (U-87 mg) as a function of the concentration of bis-*nido*-carborane dequalinium salt (48 h incubation); (B) cell viability after 48 h of incubation with boron-loaded PC or with boron-loaded DMPC liposomes; error bars are mean ± sd (*n* = 3).

Towards a preliminary assessment of the effects of DLC-carboranes on cell growth,^49^ U-87 MG cells were incubated over a specified time period with systematically varied concentrations of bis-*nido*-carborane dequalinium salt (BOR4). Cell viability data (Fig. 4) demonstrated a concentration-dependent toxicity effect and allowed the estimation of IC_50_ of this molecule at 0.832 μM. Analogous experiments with carborane-loaded PC and DMPC liposomes over the boron concentration range 0.2–4.0 μM indicated the biocompatibility of these formulations at boron concentrations of the order of ≤2.0 μM boron. A recent study has investigated the pharmacological behavior of BOR, BOR2, BOR3, BOR4 and BOR5 in malignant, cancer stem and normal cell lines. The work identified selective cytotoxic behavior towards tumor cells and cancer stem cells, while normal cells were seen to recapitulate their physiological proliferation rate upon removal of the DLC-carborane from cultures.^10^ Tested against the criterion of selective cytotoxicity behavior, the same study identified BOR2 and BOR3 as the most promising candidate materials for further investigation.^10^

## Discussion and conclusions

**Fig. 5:**
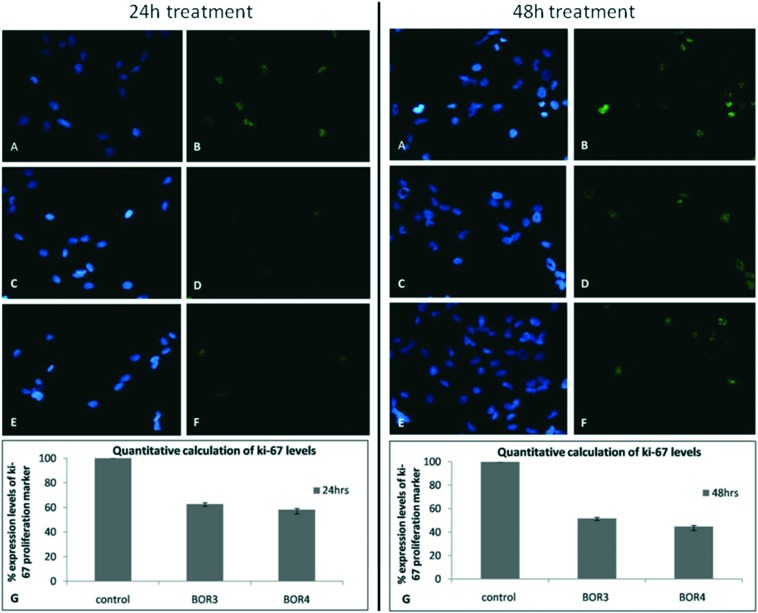
The effect of DLC-carboranes on the levels of the proliferation marker Ki-67 in primary GBM CSCs. EGFRneg primary GBM CSCs were each treated for 24 h (left) or 48 h (right) with the IC_50_ concentrations of BOR3 (7 × 10^–7^ m) or BOR4 (6 × 10^–7^ m). Panels A and B, untreated (control) cultures; panels C and D, BOR3-treated cultures; panels E and F, BOR4-treated cultures; panels A, C and E, DAPI-stained cell nuclei; panels B, D and F, Ki-67 expression levels; panel G, quantification of Ki-67 by means of the data obtained from panels B, D and F. The results are presented as a percentage of the Ki-67 expression determined for untreated control cultures and represent the mean (±sd) of two independent experiments. Statistical analysis was performed using paired *t*-test (*p* < 0.05).

While limited, studies to date show the promise of DLC-carboranes to act as BNCT agents that target cancer and primary GBM CSCs in the presence of normal cells with a high degree of selectivity.^10^,^48^ Complementary work^10^ has further revealed the capability of DLC-carboranes to effect growth inhibition in primary GBM CSCs (Fig. 5).

Underpinned by the principle that the elimination of CSCs may lead to positive long-term clinical outcomes,^50^,^51^ DLC-carboranes offer the potential for selective cytotoxicity within heterogeneous tumour cell populations. It has been shown that the mechanism of action of DLC-carboranes involves activation of the p53/p21 gene axis. It appears that the selective accumulation of DLC-carboranes to the mitochondria of tumour cells provides the stimulus that ultimately activates the tumour suppressor protein p53, which mediates the DNA damage-induced checkpoint mechanism through the trans-activation of growth inhibitory genes, such as p21. This in turn causes permanently malignant cells to enter a phase of p53-dependent G1 growth arrest, and prevents cell cycling and division. Notably, it has been shown that malignant cells exposed to DLC-carboranes simultaneously activate the expression of genes that are linked to apoptosis (*e.g.* bax, bad, caspases 3 and 9), to survival (*e.g.* bcl-2) and to mitogenesis (*e.g.* c-myc, cyclin D1, cdk4).^10^ The opposing cellular response functions in the observed gene expression profile may be explained in terms of an attempt by cells to overcome the DLC-carborane-triggered cell-cycle arrest mediated by p53/p21 through genetic manipulation.

Evidence to date suggests that DLC-carboranes may be of value not only to BNCT but also as stand-alone anticancer drugs. Consequently, DLC-carboranes represent a new class of anticancer agents, the pharmacological efficacy and safety profiles of which merit investigation with reference to their chemical structure such that the most promising compounds are identified for clinical verification. In terms of chemistry, the significance of the nature of the bond that connects the DLC and carborane moieties needs to be understood in order to allow the research focus to shift to ionic or covalent structures according to their promise to meet the performance requirements imposed by the BNCT protocol.
