# Correction: Boron-containing delocalised lipophilic cations for the selective targeting of cancer cells

**DOI:** 10.1039/c7md90004j

**Published:** 2017-02-22

**Authors:** Gianpiero Calabrese, Anis Daou, Aikaterini Rova, Eirini Tseligka, Ioannis S. Vizirianakis, Dimitrios G. Fatouros, John Tsibouklis

**Affiliations:** a School of Life Science , Pharmacy and Chemistry , Kingston University London , Penrhyn Road , Kingston-upon-Thames , Surrey , KT1 2EE , UK . Email: G.Calabrese@kingston.ac.uk; b Department of Pharmacology , School of Pharmacy , Aristotle University of Thessaloniki , GR-54124 Thessaloniki , Greece; c Department of Pharmaceutical Technology , School of Pharmacy , Aristotle University of Thessaloniki , GR-54124 Thessaloniki , Greece; d School of Pharmacy and Biomedical Sciences , University of Portsmouth , Portsmouth , PO1 2DT , UK

## Abstract

Correction for “Boron-containing delocalised lipophilic cations for the selective targeting of cancer cells” by Gianpiero Calabrese *et al.*, *Med. Chem. Commun.*, 2017, **8**, 67–72.



## 

The authors regret that on their paper the authors' names were displayed incorrectly. The correct listing is shown above.

The Royal Society of Chemistry apologises for these errors and any consequent inconvenience to authors and readers.

